# MicroRNA-130b Promotes Cell Aggressiveness by Inhibiting Peroxisome Proliferator-Activated Receptor Gamma in Human Hepatocellular Carcinoma

**DOI:** 10.3390/ijms151120486

**Published:** 2014-11-07

**Authors:** Kangsheng Tu, Xin Zheng, Changwei Dou, Chao Li, Wei Yang, Yingmin Yao, Qingguang Liu

**Affiliations:** Department of Hepatobiliary Surgery, the First Affiliated Hospital of Xi’an Jiaotong University, Xi’an 710061, China; E-Mails: xin.zheng.xjtu@gmail.com (X.Z.); dcwtmac@sina.cn (C.D.); qixinlichao@163.com (C.L.); drbobyang@163.com (W.Y.); yaoyingmin@sina.com (Y.Y.)

**Keywords:** microRNA-130b, hepatocellular carcinoma, PPAR-γ (peroxisome proliferator-activated receptor gamma), invasion, tumor metastasis

## Abstract

MircroRNA-130b (miR-130b) is proposed as a novel tumor-related miRNA and has been found to be significantly dysregulated in tumors. In this study, the expression level of miR-130b was found to be obviously higher in hepatocellular carcinoma (HCC) tissues than that in nontumor tissues. Further, miR-130b was expressed at significantly higher levels in aggressive and recurrent tumor tissues. Clinical analysis indicated that high-expression of miR-130b was prominently correlated with venous infiltration, high Edmondson-Steiner grading and advanced tumor-node-metastasis (TNM) tumor stage in HCC. Elevated miR-130b expression was observed in all HCC cell lines (HepG2, SMMC-7721, Huh7, Hep3B and MHCC97H) as compared with that in a nontransformed hepatic cell line (LO2). Furthermore, an inverse correlation between miR-130b and E-cadherin and a positive correlation between miR-130b and Vimentin were observed in HCC tissues. Down-regulation of miR-130b expression reduced invasion and migration in both Hep3B and MHCC97H cells. Peroxisome proliferator-activated receptor gamma (PPAR-γ) was inversely correlated with miR-130b expression in HCC tissues. In addition, down-regulation of miR-130b restored PPAR-γ expression and subsequently suppressed epithelial-mesenchymal transition (EMT) in HCC cells. We identified PPARγ as a direct target of miR-130b in HCC *in vitro*. Notably, PPAR-γ knockdown abolished down-regulation of miR-130b-inhibited EMT in MHCC97H cells. In conclusion, miR-130b may promote HCC cell migration and invasion by inhibiting PPAR-γ and subsequently inducing EMT.

## 1. Introduction

MicroRNAs (miRNAs) are an abundant group of endogenous non-coding single strand RNAs of ~22 nucleotides [1]. They regulate gene expression at the post-transcriptional level by translational repression or degradation of target mRNA. In this manner, they participate in various biological processes including cell development [2,3], cell cycle [4], and stem cell renewal [5]. Aberrant expression of miRNAs plays a key role in cancer development and progression through modulating oncogenic and tumor suppressor pathways. Using microarray or real-time PCR, miRNA expressing profiles which can differentiate cancer and normal tissues have been identified [6]. Moreover, different types of cancers have varied expression pattern of miRNAs. Due to their high stability in serum, miRNAs are becoming promising diagnostic and prognostic biomarkers for patients with malignancy [7,8].

miR-130b is proposed as a novel tumor-related miRNA and has been found to be significantly dysregulated in tumors [9]. It was down-regulated in papillary thyroid carcinomas [10], endometrial cancer [11], pituitary adenomas [12], and pancreatic cancer [13]. It inhibited proliferation and invasion of pancreatic cancer cells by directly modulating signal transducer and activator of transcription 3 (STAT3) [13]. Repressed expression of miR-130b by p53 mutants led to zinc-finger E-box binding homeobox 1 (ZEB1)-dependent epithelial-mesenchymal transition (EMT) in endometrial cancer [11]. However, in melanoma [14], gastric cancer [15,16], bladder cancer [17], colorectal cancer [18], and metastatic renal carcinoma [19], miR-130b was found to be up-regulated. Elevated miR-130b in plasma was associated with poor response of chemotherapy in colorectal cancer [20]. Therefore, the functional significance of miR-130b in cancer development and progression seem to be cancer-type specific. Recently, miR-130b was identified as a robust biomarker of hepatocellular carcinoma (HCC) with high positive predictive value [21]. It was reported to be elevated in HCC tissues [22] and in patients’ serum [21]. And compared with CD133-cells with lower expression of miR-130b, CD133+ cells, which were identified as tumor-initiating cells of HCC, had higher expression of miR-130b and thereby possessed a greater ability to form undifferentiated tumor spheroids [23]. These reports indicate that miR-130b acts as an onco-miRNA in HCC. However, the molecular pathways through which miR-130b modulates the development and progression of HCC have not been elucidated.

In this study, we demonstrate that elevated miR-130b expression is observed in the aggressive phenotype of HCC. The high-expression of miR-130b is associated with poor prognostic features in HCC. The expression level of miR-130b is correlated with EMT markers in HCC tissues. Down-regulation of miR-130b inhibits HCC cell migration and invasion *in vitro*. Furthermore, miR-130b is inversely correlated with PPAR-γ in HCC tissues and it directly regulates PPAR-γ abundance in HCC cells. The effect of miR-130b down-regulation on EMT is blocked by PPAR-γ knockdown in MHCC97H cells. Our results demonstrate that miR-130 potentiates the invasive behavior of HCC cells and may contribute to tumor metastasis by inhibiting PPAR-γ and promoting EMT.

## 2. Results

### 2.1. Elevated miR-130b Expression Confers Metastasis and Recurrence of HCC (Hepatocellular Carcinoma)

**Figure 1:**
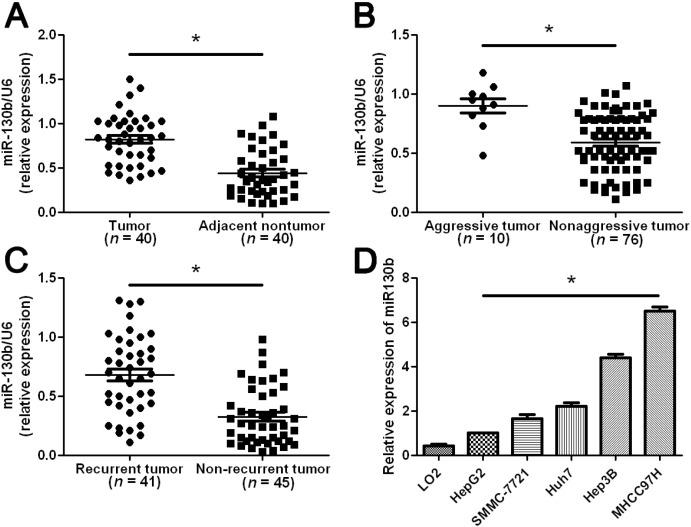
The expression levels of miR-130b in HCC (Hepatocellular Carcinoma) tissues and cells. Comparing differences in the expression levels of miR-130b between (**A**) HCC and matched nontumor tissues; (**B**) aggressive and nonaggressive tumor tissues; (**C**) HCC tissues arising from recurrent and non-recurrent groups; and (**D**) HCC cell lines with different metastatic potentials and the immortalized hepatic cell line LO2. Values are depicted as Mean ± SEM; * *p* < 0.05 by *t* test.

We tested the expression of miR-130b by qRT-PCR and normalized against an endogenous control (U6 RNA) in 40 pairs of randomly selected tumor tissues and matched adjacent nontumor tissues from HCC patients who received liver resection. The expression level of miR-130b in HCC tissues was significantly higher than that in matched adjacent nontumor liver tissues (*p* < 0.05, Figure 1A). HCC cases that showed intrahepatic spreading, venous infiltration or tumor invasion into bile ducts were considered as aggressive HCC tissues. As compared with nonaggressive HCC tissues, miR-130b levels were prominently up-regulated in aggressive HCC tissues (*p* < 0.05, Figure 1B). Furthermore, miR-130b levels were obviously increased in tumor tissues arising from patients with intrahepatic tumor recurrence or extrahepatic metastasis as compared with those in tumor tissues arising from patients without tumor recurrence (*p* < 0.05, Figure 1C). Thus, up-regulation of miR-130b level was correlated with metastasis and recurrence of HCC.

Next, we analyzed miR-130b expression in a nontransformed hepatic cell line (LO2) and a panel of HCC cell lines (HepG2, SMMC-7721, Huh7, Hep3B and MHCC97H). The miR-130b expression was significantly up-regulated in all HCC cell lines as compared with that in LO2 (*p* < 0.05, Figure 1D). Moreover, miR-130b expression in the highly metastatic HCC cell line MHCC97H was obviously higher than those in the low metastatic HCC cell lines including HepG2, SMCC-7721, Huh7 and Hep3B (Figure 1D). These data indicates that elevated miR-130b expression confers increased metastatic potential of HCC cells.

### 2.2. Clinical Significance of miR-130b Expression in HCC Specimens

Eighty-six samples of HCC tissues were subjected to qRT-PCR for miR-130b expression. We determined 0.49 (mean level of miR130b) as a cutoff value for the expression level of miR-130b. The expression of miR-130b was considered as either low (<0.49, *n* = 46) or high (≥0.49, *n* = 40). As shown in Table 1, the high-expression of miR-130b was prominently associated with venous infiltration (*p* = 0.009), high Edmondson-Steiner grading (*p* = 0.008) and advanced TNM tumor stage (*p* < 0.001). Thus, our results indicate that high-expression of miR-130b is correlated with malignant clinicopathologic characteristics in HCC.

**Table 1:** Correlation between the clinicopathologic characteristics and miR-130b expression in HCC.

Characteristics		Total No. of Patients, *n* = 86	No. of Patients		*p*
			miR-130b ^high^	miR-130b ^low^	
Age (year)	<50	36	19	17	0.323
	≥50	50	21	29	
Sex	Male	65	28	37	0.261
	Female	21	12	9	
HBV	Absent	28	9	19	0.063
	Present	58	31	27	
Serum AFP level (ng/mL)	<400	32	14	18	0.693
	≥400	54	26	28	
Tumor size (cm)	<5	32	16	16	0.618
	≥5	54	24	30	
No. of tumor nodules	1	68	28	40	0.054
	≥2	18	12	6	
Cirrhosis	Absent	36	13	23	0.101
	Present	50	27	23	
Venous infiltration	Absent	76	31	45	0.009 *
	Present	10	9	1	
Edmondson-Steiner grading	I + II	65	25	40	0.008 *
	III + IV	21	15	6	
TNM tumor stage	I + II	66	22	44	<0.001 *
	III + IV	20	18	2	

HCC, hepatocellular carcinoma; HBV, hepatitis B virus; AFP, alpha-fetoprotein; TNM, tumor-node-metastasis; * Statistically significant.

### 2.3. High-Expression of miR-130b Correlates with Mesenchymal Phenotype of HCC

**Figure 2:**
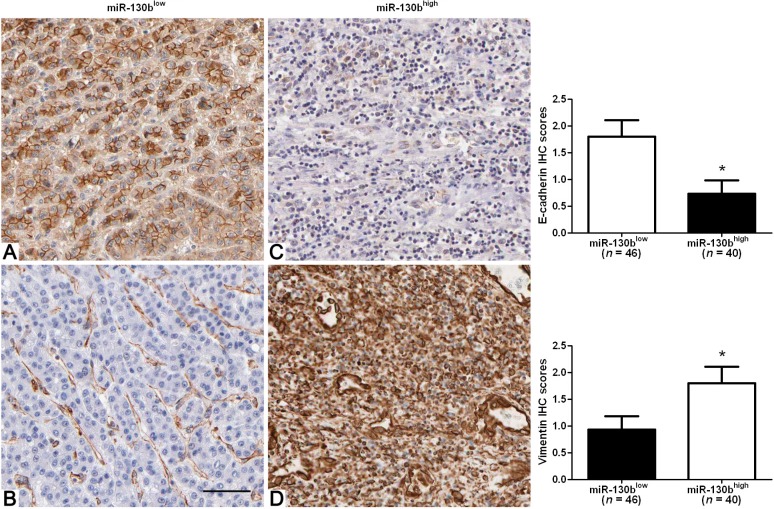
Immunohistochemical analysis of E-cadherin and Vimentin in HCC samples. In cases of low miR-130b expression (**A**,**B**); there was strong E-cadherin and no detectable Vimentin protein expression in the same tissue section. In contrast, in the case of high miR-130b expression (**C**,**D**), there was no detectable E-cadherin and strong Vimentin protein expression. Values are depicted as Mean ± SEM; * *p* < 0.05 by *t* test. Scare bar = 100 μm.

Since EMT is the leading mechanism involved in HCC metastasis and characterized by loss of cell polarity and intracellular junctions and acquirement of mesenchymal features [24]. We next analyzed the correlation among miR-130b expression, E-cadherin expression and Vimentin expression in 86 HCC cases. We found that the expression level of E-cadherin in low miR-130b expression group was significantly higher than that in high miR-130b expression group (*p* < 0.05, Figure 2). Spearman correlation analysis indicated an inverse correlation between the expression of miR-130b and E-cadherin (*rho* = −0.4920, *p* = 0.015). Furthermore, the expression level of Vimentin in the low miR-130b expression group was significantly lower than that in high miR-130b expression group (*p* < 0.05, Figure 2). A positive relationship between the expression of miR-130b and Vimentin was observed in the same cohort of HCC cases (*rho* = 0.4590, *p* = 0.037). Thus, the up-regulation of miR-130b may be responsible for the progression of EMT in HCC.

### 2.4. Promoting Effect of miR-130b on HCC Cell Migration and Invasion

**Figure 3:**
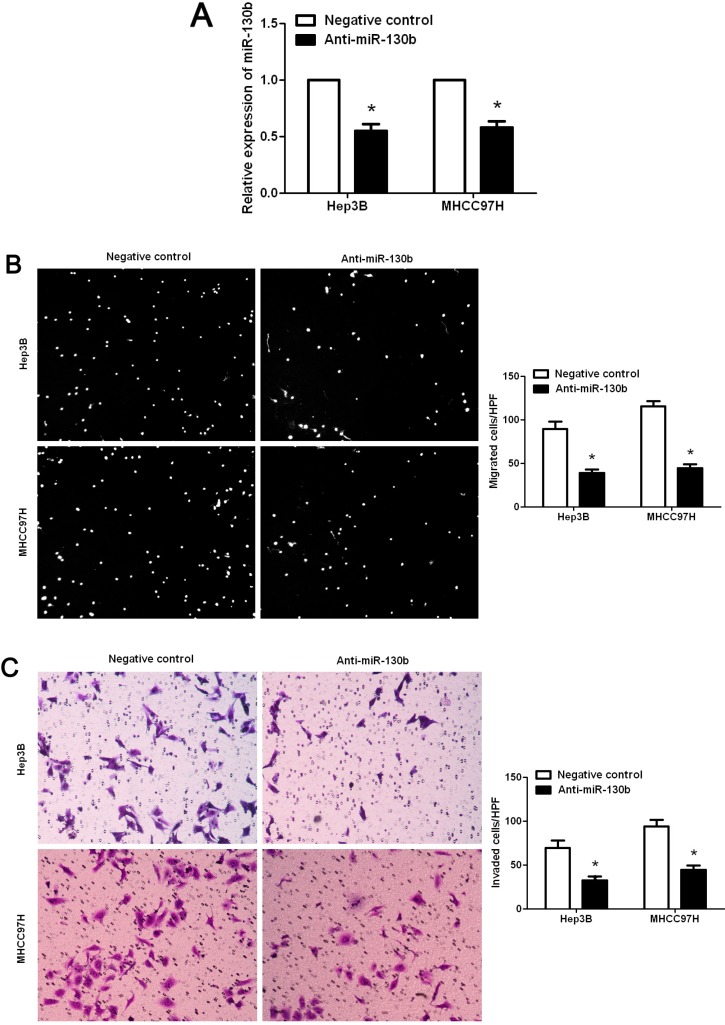
MiR-130b regulates migration and invasion of HCC cells. (**A**) Hep3B and MHCC97H cells that were transfected with miR-130b inhibitors (anti-miR-130b) and negative control, respectively, were subjected to qRT-PCR for miR-130b. *n* = 6; * *p* < 0.05 by *t* test; (**B**) Cell migration as measured by Boyden chamber assays was inhibited by down-regulation of miR-130b in Hep3B and MHCC97H cells as compared with control cells. *n* = 6 repeats with similar results, * *p* < 0.05 by *t* test; and (**C**) MiR-130b down-regulating Hep3B and MHCC97H cells conferred a lowernumber of invaded cells as compared with control cells. *n* = 6 repeats with similar results; * *p* < 0.05 by *t* test. Values are depicted as Mean ± SEM.

To investigate the role of miR-130b in HCC, we suppressed the expression level of miR-130b in two HCC cell lines, Hep3B and MHCC97H. As assessed by qRT-PCR, the expression of miR-130b was down-regulated by ectopically expressing miR-130b inhibitors in both cell lines (*p* < 0.05, respectively, Figure 3A). Boyden chamber assays were performed to test the effect of altering miR-130b levels on HCC cell migration. We found that down-regulation of miR-130b led to a significant reduction of cell migration in both Hep3B and MHCC97H cells (*p* < 0.05, respectively, Figure 3B). Furthermore, as determined by Transwell assays, the number of invaded Hep3B and MHCC97H cells was significantly reduced after down-regulation of miR-130b (*p* < 0.05, respectively, Figure 3C). Thus, miR-130b exerts a pro-metastatic effect on HCC.

### 2.5. MiR-130b Promotes EMT (Epithelial-Mesenchymal Transition) by Inhibiting PPARγ in HCC

**Figure 4:**
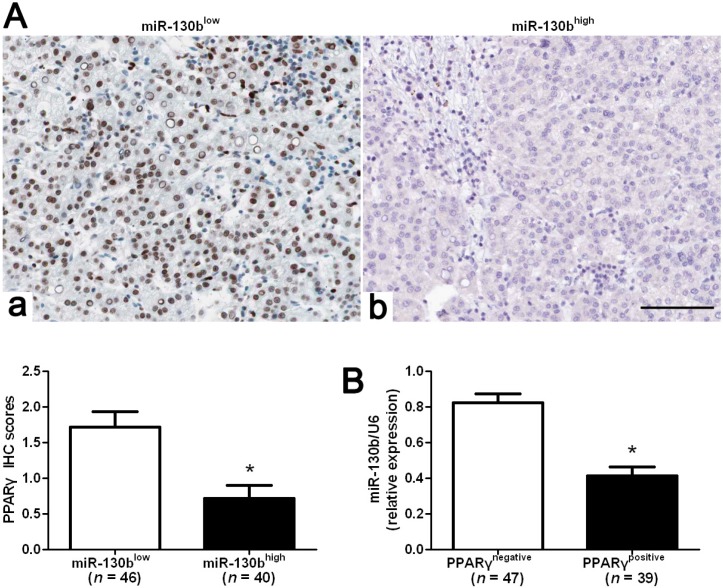
Correlation between miR-130b and PPAR-γ in HCC tissues. (**A**) In cases of low miR-130b expression (**a**), there was strong PPAR-γ protein expression in the same tissue section. In contrast, in the case of high miR-130b expression (**b**), there was no detectable PPAR-γ protein expression; (**B**) The expression level of miR-130b in the positive PPAR-γ expression group was significantly lower than that in the negative PPAR-γ expression group. Values are depicted as the mean ± SEM; * *p* < 0.05 by *t* test. Scare bar = 100 μm.

**Figure 5:**
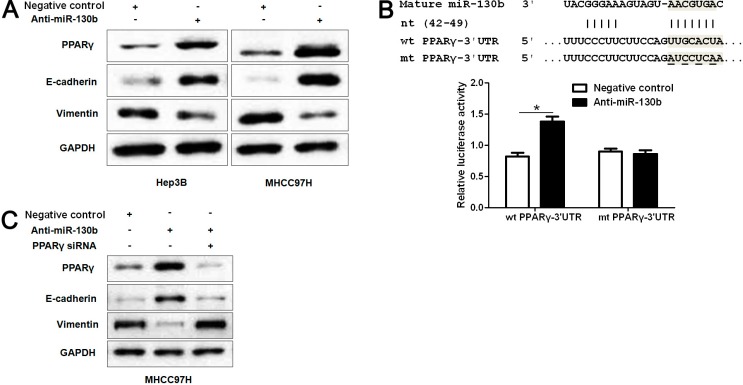
MiR-130b promotes EMT by suppressing PPAR-γ expression in HCC cells. (**A**) Hep3B and MHCC97H cells that were transfected with miR-130b inhibitors (anti-miR-130b) and negative control, respectively, were subjected to western blot (WB) for PPAR-γ, E-cadherin and Vimentin. Representative WB showed that down-regulation of miR-130b obviously increased protein expression of PPAR-γ and E-cadherin and reduced Vimentin expression in HCC cells; (**B**) miR-130b and its putative binding sequence in the 3'-UTR of PPAR-γ. The mutant miR-130b binding site was generated in the complementary site for the seed region of miR-130b (wt, wild type; mt, mutant type). Anti-miR-130b led to a noticeable increase in luciferase activity of wt 3'-UTR of PPAR-γ in MHCC97H cells. Data were normalized by the ratio of firefly and Renilla luciferase activities measured at 48 h post-transfection. *n* = 3 repeats with similar results. * *p* < 0.05; and (**C**) Representative WB showed that PPAR-γ knockdown abolished the expression change of PPAR-γ, E-cadherin and Vimentin induced by miR-130b suppression in MHCC97H cells. Data are representative of multiple repeats with similar results.

Previous studies reported that miR-130b led to PPARγ suppression that in turn promotes EMT progression in colorectal cancer [18]. We evaluated the correlation between PPARγ and miR-130b expression in our HCC samples. The expression of PPARγ levels in miR-130b high-expressing tumors was significantly lower than those in miR-130b low-expressing tumors (*p* < 0.05, Figure 4A). Furthermore, PPARγ immunoreactivity was considered as either negative (score 0) or positive (scores 1 to 3). According to PPARγ status, PPARγ negative-expressing tumors (*n* = 47) showed higher levels of miR-130b as compared with PPARγ positive-expressing ones (*n* = 39, *p* < 0.05, Figure 4B). Spearman correlation analysis indicated that miR-130b was inversely correlated with PPARγ expression in HCC (*rho* = −0.6216, *p* < 0.001). Next, Hep3B and MHCC97H cells that were transfected with miR-130b inhibitors or negative control were subjected to western blot for PPARγ, E-cadherin and Vimentin. As assessed by immunoblotting, down-regulation of miR-130b obviously restored the expression of PPARγ protein and led to up-regulation of E-cadherin and down-regulation of Vimentin in both Hep3B and MHCC97H cells (Figure 5A). To further demonstrate that PPARγ is directly targeted by miR-130b in HCC cells, we investigated whether the miR-130b directly interacted with the 3'-UTR of PPARγ mRNA using a dual-luciferase reporter assay. As expected, inhibition of endogenous miR-130b by miR-130b inhibitors led to increased luciferase activity of the wild-type reporter but not the mutant reporter (*p* < 0.05, Figure 5B). Furthermore, PPARγ knockdown by a specific siRNA abrogated the expression change of these genes induced by down-regulation of miR-130b in MHCC97H cells (Figure 5C). Taken together, these data indicate that miR-130b directly suppresses PPARγ expression that subsequently promotes EMT progression in HCC.

## 3. Discussion

HCC is the fifth most frequent malignancy and the third leading cause of cancer-related death worldwide, accounting for more than 500,000 deaths every year [25]. Increasing studies have reported that miRNAs regulated hepatocarcinogenesis-related gene expression, indicating a new insight in the initiation and progression of HCC [26]. Recently, miR-130b was identified as a robust biomarker of HCC with high positive predictive value [21]. It was reported to be elevated in HCC tissues and in patients’ serum [21,23]. We initially detected the expression level of miR-130b in 40 pairs of HCC tissues and adjacent nontumor tissues. Quantification of the data indicated that miR-130b expression in tumor tissues was significantly up-regulated as compared with that in nontumor tissues. Furthermore, miR-130b was expressed at significantly higher levels in aggressive tumors than in nonaggressive tumors. Importantly, our results showed that elevated miR-130b expression conferred a significant higher recurrence rate for HCC patients. Clinical analysis found that miR-130b was expressed at significantly higher levels in HCC patients with venous infiltration, high Edmondson-Steiner grading and advanced TNM tumor stage, suggesting that high-expression of miR-130b is obviously correlated with poor prognostic features in HCC. Recently, Wang *et al.* [27] reported that miR-130b expression level was significantly higher in HCC tissues compared with normal adjacent liver tissues and high expression of miR-130b correlated with poor prognosis of HCC patients. Altogether, these results suggest that miR-130b is critical for prognosis determination in HCC patients. Moreover, elevated miR-130b expression was observed in HCC cell lines, especially in the highly metastatic HCC cell line MHCC97H. Our results indicate that miR-130b might be critical for the regulation of tumor invasion and metastasis in HCC patients.

EMT, a dynamic and reversible cellular process, is characterized by loss of cell polarity and intracellular junctions and acquirement of mesenchymal features, resulting in increased HCC cell migration and invasion [24]. Thus, we determined the expression of epithelial marker (E-cadherin) and mesenchymal marker (Vimentin) in HCC samples with either low or high miR-130b expression. Interestingly, we found that the expression level of E-cadherin in low miR-130b expression group was significantly higher than that in the high miR-130b expression group. Furthermore, an inverse correlation between miR-130b and E-cadherin expression was observed in HCC tissues. In contrast, Vimentin was expressed at significant lower levels in low miR-130b expression group as compared with that in high miR-130b expression group. Moreover, miR-130b was positively associated with Vimentin expression in HCC tissues. These data indicate that miR-130b expression level may be correlated with the EMT of HCC. Our loss-of-function experiments demonstrated that down-regulation of miR-130b expression significantly reduced the number of migrated and invaded cells in both Hep3B and MHCC97H cells.

Peroxisome proliferator-activated receptor-γ (PPAR-γ) is a transcription factor controlling cell proliferation, differentiation and apoptosis [28] and has been found to be downregulated in HCC [29]. Ectopic expression of PPAR-γ inhibited metastatic activity of HCC cells [30]. Recently, PPAR-γ in colorectal cancers has been found to be a direct target of miR-130b and contribute to EMT mediated by miR-130b [18]. In our study, the inverse correlation between miR-130b and PPAR-γ expression was observed in HCC tissues. Furthermore, we investigated the regulatory effect of miR-130b on PPAR-γ, E-cadherin and Vimentin *in vitro*. Our data showed that down-regulation of miR-130b increased the expression level of PPAR-γ and suppressed EMT in two different HCC cell lines, Hep3B and MHCC97H. Herein, we validated PPARγ as a direct functional target of miR-130b in HCC, adding information to previously reported cell types [18,31]. However, the putative binding sequences of miR-130b were not found in the 3'-UTR of E-cadherin and Vimentin. Notably, the regulatory effect of reduced miR-130b expression on PPAR-γ, E-cadherin and Vimentin were inverted by PPAR-γ siRNA in MHCC97H cells. Thus, miR-130b may promote EMT by inhibiting PPAR-γ expression in HCC.

In conclusion, we find that miR-130b is up-regulated in HCC tissues, especially in aggressive and recurrent tumor tissues. The high-expression of miR-130b is evidently correlated with poor prognostic features in HCC. Otherwise, the expression level of miR-130b is correlated with EMT markers in HCC. We demonstrate that down-regulation of miR-130b reduces cell migration and invasion by restoring PPAR-γ expression and subsequently suppressing EMT in HCC cells. Taken together, we consider that miR-130b may potentially act as an onco-miRNA, and may also be a therapeutic target, in HCC.

## 4. Experimental Section

### 4.1. Ethical Review

The Xi’an Jiaotong University Ethics Committee approved all protocols according to the Helsinki Declaration (as revised in Tokyo 2004) and informed consent was signed by each patient. All animal protocols were approved by the Institutional Animal Care and Use Committee of Xi’an Jiaotong University.

### 4.2. Clinical Samples

Eighty-six HCC samples were collected from patients including 69 males and 17 females, who underwent the resection of their primary HCC in the Department of Hepatobiliary Surgery at the First Affiliated Hospital of Xi’an Jiaotong University during January 2006 to December 2008, with a median follow-up time of 29 months. The clinicopathological data are shown in Table 1. All samples were used after obtaining informed consent. Patients did not receive preoperative chemotherapy or embolization. In these cases, ten patients had extrahepatic metastasis and 41 patients had tumor recurrence.

### 4.3. Immunohistochemical Staining

Immunohistochemistry was performed on paraformaldehyde-fixed paraffin sections. E-cadherin (24E10, #3195; Cell Signaling, Beverly, MA, USA) (1:400), Vimentin (C-20, sc-7557; Santa Cruz Biotechnology, Santa Cruz, CA, USA) (1:200) and PPAR-γ (C26H12, #2435; Cell Signaling) (1:400) antibodies were used in immunohistochemistry with streptavidin peroxidase conjugated (SP-IHC). Immunohistochemistry was performed as previously reported [32]. The percentage of positive tumor cells was graded as per the following criteria: 0, less than 10%; 1, 10%–30%; 2, 31%–50%; 3, more than 50%.

### 4.4. Cell Lines and Transfection

The human immortalized normal hepatocyte cell line, LO2, and five HCC cell lines, HepG2, Hep3B, SMMC-7721, MHCC97H and Huh7 (the Institute of Biochemistry and Cell Biology, Chinese Academy of Sciences, Shanghai, China), were cultured in complete Dulbecco’s modified Eagle medium (DMEM, Gibco, Grand Island, NY, USA) containing 10% fetal bovine serum (FBS, Gibco) with 100 units/mL penicillin and 100 μg/mL streptomycin (Sigma, St-Louis, MO, USA) in a humidified incubator at 37 °C containing 5% CO_2_.

MiRNA vectors, including miR-130b inhibitor and the negative control for the miR-130b inhibitor, and PPAR-γ siRNA (5'-AAUAUGACCUGAAGCUCCAAGAAUAAG-3') were purchased from Genecopoeia (Guangzhou, China). Cells were transfected with the vectors and siRNA mentioned above using Lipofectamine 2000 according to the manufacturer’s instructions (Invitrogen, Carlsbad, CA, USA).

### 4.5. Western Blot

The following primary antibodies were used in the immunoblotting assays: PPAR-γ (1:1000), E-cadherin (1:1000), Vimentin (1:1000) and GAPDH (G8140; US Biological, Swampscott, MA, USA) (1:5000). Horseradish peroxidase-conjugated goat anti-mouse or anti-rabbit secondary antibodies (Bio-Rad, Hercules, CA, USA) were used at a 1:1000–1:5000 dilution and detected using a Western Blotting Luminol Reagent (sc-2048; Santa Cruz Biotechnology), as described in our previous study [33].

### 4.6. Boyden Chamber and Transwell Assays

A Boyden chamber assay (NeuroProbe, Gaithersburg, MD, USA) was used to analyze HCC cell migration as previously described [34]. Transwell assays were done in 6 well plates with Transwell inserts equipped with 8-μm pores (Nalge Nunc International Corp, Naperville, IL, USA) coated with Matrigel at 1:6 dilution (Becton Dickinson Labware, Bedford, MA, USA) as previously described [35].

### 4.7. Real Time Quantitative Reverse Transcription-PCR (qRT-PCR)

The PCR amplification for the quantification of the miR-130b and U6 was performed using TaqMan miRNA Reverse Transcription Kit (Applied Biosystms, Foster City, CA, USA) and TaqMan Human MiRNA Assay Kit (Applied Biosystems). The relative expression of miR-130b was shown as fold difference relative to U6.

### 4.8. Luciferase Reporter Assay

The predicted 3'-UTR sequence of PPARγ that interacted with miR-130b, together with a corresponding mutated sequence within the predicted target sites, were synthesized and inserted into the pRL-TK control vector (Promega, Madison, WI, USA). MHCC97H cells that were seeded in a 96-well plate were transfected with 120ng miR-130b inhibitor or negative control. Cells were cotransfected with 30 ng of the wild-type or mutant 3'-UTR of PPARγ mRNA. Transfections were performed using 0.45 μL of Fugene (Promega). 48 h after transfection, cells were collected and measured according to the manufacturer’s instructions (Dual-Luciferase Assay System; Promega). pRL-TK expressing Renilla luciferase was cotransfected as an internal control to correct the differences in both transfection and harvest efficiencies.

### 4.9. Statistical Analysis

Results are expressed as Mean ± SEM. Significance was established, with the SPSS statistical package for Windows Version 13 (SPSS, Chicago, IL, USA) and GraphPad Prism 5 software (GraphPad Software, Inc, San Diego, CA, USA), using a Pearson chi-squared test, a Spearman’s rank correlation coefficient or a two-tailed Student’s *t* test when appropriate. Difference were considered significant when *p* < 0.05.

## 5. Conclusions

In conclusion, this study shows that the expression level of miR-130b is up-regulated in tumor tissues as compared with matched adjacent nontumor liver tissues and that high miR-130b expression level confers metastasis and recurrence of HCC. Clinical analysis indicated that high-expression of miR-130b was prominently correlated with poor clinicopathological parameters in HCC. Elevated miR-130b expression levels are observed in HCC cell lines, especially in the highly metastatic cell lines. Furthermore, miR-130b is correlated with EMT markers, E-cadherin and Vimentin, in HCC tissues. Functional studies demonstrate that down-regulation of miR-130b inhibits HCC cell migration and invasion. An inverse correlation between miR-130b and PPAR-γ expression is observed in HCC tissues. Importantly, down-regulation of miR-130b increases PPAR-γ expression and subsequently suppressed EMT in HCC cells. PPAR-γ is identified as a direct target of miR-130b in HCC. PPAR-γ knockdown can abolish the effect of miR-130b down-regulation on anti-metastasis in HCC, suggesting that miR-130b functions as a pro-metastatic factor by downregulating PPAR-γ. This study reveals that miR-130b may play a critical role in the invasion and metastasis of HCC.
